# The European & Developing Countries Clinical Trials Partnership (EDCTP) Knowledge Hub: developing an open platform for facilitating high-quality clinical research

**DOI:** 10.1186/s13063-022-06311-y

**Published:** 2022-05-07

**Authors:** Samuel Driver, Shan Gray, Welile Sikhondze, Ken Awuonda, Helena Wilcox, Alexis Segrt, Lara Pandya, Johanna Roth, Michael Makanga, Trudie Lang

**Affiliations:** 1 Centre for Tropical Medicine and Global Health, Nuffield Department of Medicine, University of Oxford, Oxford, OX3 7FZ UK; 2 National TB Control Program, Ministry of Health, Mbabane, Eswatini; 3 European & Developing Countries Clinical Trials Partnership, The Hague, The Netherlands

**Keywords:** Capacity development, Research resources, Research methodology, Research leadership, African research leaders

## Abstract

There is stark global inequity in health research in terms of where studies happen, who leads the research and the ultimate beneficiaries of the results generated. Despite significant efforts made, limited research ideas are conceptualised and implemented in low-resource settings to tackle diseases of poverty, and this is especially true in sub-Saharan Africa. There is strong evidence to show that the barriers to locally led research do not vary largely between disease, study type and location and can be largely solved by addressing these common gaps. The European & Developing Countries Clinical Trials Partnership (EDCTP) was established in 2003 as a European response to the global health crisis caused by the three main poverty-related diseases HIV, tuberculosis and malaria. EDCTP has established a model of long-term sustainable capacity development integrated into clinical trials which addresses this lack of locally led research in sub-Saharan Africa, supporting the development of individual and institutional capacity and research outputs that change the management, prevention and treatment of poverty-related and neglected infectious diseases across Africa. In recognition of emergent data on what the barriers and enablers are to long-term, sustainable capabilities to run studies, EDCTP formed a new collaboration with The Global Health Network (TGHN) in September 2017, with the aim to make a set of cross-cutting tools and resources to support the planning, writing and delivery of high-quality clinical trials available to research staff wherever they are in the world, especially those in low- and middle-income countries (LMICs) via TGHN platform. These new resources developed on the ‘EDCTP Knowledge Hub’ are those identified in the mixed method study described in this commentary as being key to addressing the gaps that the research community report as the most limiting elements in their ability to design and implement studies. The Knowledge Hub aims to make these tools freely available to any potential health research team in need of support and guidance in designing and running their own studies, particularly in low-resource settings. The purpose is to provide open access to the specific guidance, information and tools these teams cannot otherwise access freely. Ultimately, this will enable them to design and lead their own high-quality studies addressing local priorities with global alignment, generating new data that can change health outcomes in their communities.

**Supplementary Information:**

The online version contains supplementary material available at 10.1186/s13063-022-06311-y.

## Background

As a major funder of clinical trials, the European & Developing Countries Clinical Trials Partnership (EDCTP) supports collaborative research that accelerates the development of accessible, suitable and affordable medical interventions to identify, treat and prevent poverty-related infectious diseases in sub-Saharan Africa, with a strong emphasis on supporting the capacity building of African researchers [1, 2]. Over the past 18 years, EDCTP has made substantial efforts to address clinical research capacity gaps and its grant funding, and other activities are contributing to the development of high-quality and sustainable health research systems in the region [3]. This includes the establishment of four EDCTP Regional Networks of Excellence, to strengthen regional networking and provide platforms for research capacity building in areas such as infrastructure development, training and mentoring, resource sharing and harmonisation [4]. EDCTP has also established a comprehensive fellowship programme providing personal support spanning all career stages to build human capacity in sub-Saharan Africa [5, 6], further complemented by the EDCTP Alumni Network and its associated web platform which encourages continued networking, collaboration and mentoring [7, 8]. Long-term sustainable research capacity development remains integrated into the EDCTP research funding strategy, in order to strengthen the enabling environment for conducting high-quality clinical research by African scientists in sub-Saharan Africa [1].

Despite the efforts of EDCTP and other major funders of health research in Africa to promote local ownership and leadership, too few studies are led from Africa, and there is a need to address this inequity [9, 10]. There is significant evidence that limited skills, knowledge and tools prevent local teams from conceptualising and implementing their own studies [9, 11–13], and importantly, our evidence shows that the challenges are cross-cutting and do not vary significantly between disease areas and types of study (De la Horra A, Feune de Colombi N, Baker B et al: An evidence-led Essential Research Skills Training Curriculum: the outcome of an international and multidisciplinary Delphi consensus study, in preparation). EDCTP and The Global Health Network (TGHN) [14] have formed a new partnership to develop the ‘EDCTP Knowledge Hub’ (https://edctpknowledgehub.tghn.org/), which aims to provide researchers involved in clinical trials, non-interventional studies and product-focused implementation research in all low-resource settings with access to relevant tools and guidance, a lack of which is known to be a significant obstruction in undertaking high-quality research in all types of studies [15]. So far, this hub has a Protocol Development Toolkit, a Data Management Portal and a Data Sharing Toolkit. Ultimately, this Knowledge Hub aims to support research teams right through the whole process, from setting a research question, turning this into a robust protocol, setting up and running a quality study by implementation of this protocol in their clinical setting, through to managing, analysing and sharing their data and translating the findings into policy and practice. Whilst developed to support EDCTP grant holders and applicants, these online, open-access and free resources are available to improve the quality of the protocols, assure data quality and reliability and support awareness and capacity development around data sharing, for all health research teams wherever they are in the world, especially those in low-resource settings.

## Why develop a Knowledge Hub to provide research tools?

The development of a robust protocol and data management plan is essential for ensuring the validity and quality of data in all types of clinical research and are essential precursors for data sharing. There is currently no ‘one-stop shop’ for protocol development, data management and data sharing, and there is a lack of knowledge about what exists or what is needed in order to conduct good research [9, 11–13]. Data management is often grossly overlooked, poorly supported and badly performed [16–18], and though data sharing is now a standard requirement by publishers, funders and regulatory agencies, there is a lack of clear guidance on when, where and how to actually share data [19]. Of particular relevance to EDCTP and TGHN are the challenges in study planning and sharing data faced by researchers in LMICs. To maximise the potential of data sharing and address the inequities that exist between high-income countries (HICs) and LMICs with regard to ownership and reuse, the aim is to ensure that all researchers have the awareness, skills and access to the right information to both share and access shared data globally [20].

Developing a protocol is a challenging task, whereby a question needs to be turned into a set of operational activities that will enable the right participants to be enrolled, the intervention to be given and for the right measurements to be made to determine the answer to the question set. All this needs to happen safely, ethically and accurately. Often, a protocol stems from a funding application and might be developed by a large group of people, often distanced from the clinical setting and research team who will be implementing the study. These factors can contribute to a poorly designed study. A tool kit is therefore needed to help ensure that the appropriate measurements are taken for the setting and population concerned, which measure the right outcome to answer the question. Such a toolkit would need to outline the steps and procedures required to ensure the protocol is valid, safe and ethical.

By building an online, open-access Knowledge Hub which brings together all the available relevant resources regarding protocol development, data management and data sharing, including but not limited to online training, we aim to support clinical research capacity building efforts for researchers, especially those in LMICs, who may otherwise experience difficulties in accessing the training necessary to plan and implement high-quality clinical research as well as sharing the results and data emanating from this global health research.

## Addressing the gaps

TGHN has been working with researchers in LMICs for over 10 years and has been collecting data on where the areas of difficulties lie for research teams [13, 14]. This platform already supports a strong community of practice of research teams and provides online training and resources. This partnership with EDCTP aims to strengthen our impact by combining efforts to share expertise between disease areas, organisations, teams and regions.

In order to establish the true gaps and assess the tools that already exist, a mixed-method study was undertaken to consider these three areas of difficulty (protocol development, data management and data sharing). DEVONagent Pro was utilised to carry out an Internet-based meta-search querying multiple search engines simultaneously [21]. Firstly, using search terms which might be entered by a researcher trying to gather information, such as ‘how to write a protocol’ or ‘data sharing’, followed by more in-depth three-level queries, such as ‘data management, good clinical practice, health research’. For more detail on the specific steps taken and specific search terms see Additional files 1 and 2. This identification of resources was followed by a quality assurance exercise and an exploration of the perceived gaps in content. The suitability of materials was determined initially from the summary information and subsequently by exploring the main content. Resources were first reviewed for relevance to the audit remit. Those resources clearly unrelated to clinical research could be excluded based on summary information, e.g. music videos, materials relating to Internet-based protocols or clinical care protocols. The main content of the remaining resources was reviewed using the exclusion criteria detailed in Table 1.

**Table 1:** Exclusion criteria: the specific exclusion criteria for materials

- Materials that do not fit into at least one of the pre-determined themes	
- Materials that are not open access	
- Materials that have a cost implication/charge an access fee	
- Materials that are not from a reputable organisation or course provider, e.g. an unaccredited blog or materials based on a personal or biassed opinion	
- Materials that are not in a suitable format	

These data were compiled and presented as a report at a workshop attended by over 40 attendees of the Ninth EDCTP Forum (17–21 September 2018) in Lisbon, Portugal. The EDCTP Forum has become a prominent cross-disease and inter-disciplinary conference. It brings together scientists, policymakers, funders and global health partners in the field of clinical research and development (R&D) for poverty-related diseases providing a platform to share new results and form new collaborative links with international colleagues. This Ninth EDCTP Forum was held in partnership with the Portuguese Foundation for Science and Technology and the Calouste Gulbenkian Foundation with the theme ‘Clinical research and sustainable development in sub-Saharan Africa: the impact of North-South partnerships’. It was attended by a global audience. The structured session sought feedback from the attendees on these findings, asked specifically if they were relevant to them and identified their teams’ training needs in protocol development, data management and data sharing. The findings of the workshop supported the data and so validated these findings; thus, we are confident that we have a solid overview on where the gaps lie between the training that is needed for research teams and what resources already exist.

During 2019 and 2020, 9 online surveys and 2 workshops were undertaken by TGHN and the Special Programme for Research & Training in Tropical Diseases (WHO-TDR) [22] to determine knowledge gaps within research teams, laboratories and healthcare settings. The surveys generated 7167 responses from across 153 countries. These findings have been brought together within a formal DELPHI survey that is currently being written up for publication (De la Horra A, Feune de Colombi N, Baker B et al: An evidence-led Essential Research Skills Training Curriculum: the outcome of an international and multidisciplinary Delphi consensus study. In preparation) and are being taken forward by WHO-TDR to develop a core curriculum for health research. Data management came out in every survey and workshop as missing skills and knowledge gap and was highlighted as a key skill area within the work going forward with WHO-TDR.

In the analysis, across Africa, 53.7% of respondents expressed ‘no experience’ or ‘minimum experience’ in the following:

- ‘Use of database software to find records, sort, review, edit, print and other data related functions’
- ‘Create and maintain data in a clinical data management system/database, e.g. MS Access or other database software’

Across Latin America, the most demanded training needs included ‘data analysis plans’ (54.1%) and ‘project management’ (50.5%).

The scoping exercise and workshops highlighted the degree to which research teams struggle to locate through a simple web search adequate guidance, resources and training to design protocols, set up good data management processes and share their data. Whilst more resources were discovered using more extensive searches, using specialist software, we concluded that most research teams were unlikely to be able to carry out such searches, and therefore, there is a need to make what is out there discoverable and so bring what exists together in an easy-to-use hub. Our other finding was that teams benefit from being shown resources, standards and guidance that they otherwise would not have known even existed, for example, standard outcome measures or data management terms. Availability of and easy access to these resources improve research quality and standards, remove duplication and stand to deliver better and more applicable, sharable evidence.

## Protocol Development Toolkit

During the scoping exercise, few open-access online training courses/materials were retrieved, and none appeared to be specific for low-resource settings. Many resources described as toolkits tended to be repositories of useful links to resources such as SPIRIT Statement and ICH GCP guidance [23, 24], protocol templates and SOPs rather than ‘how to’ guides with advice and examples. Guides are available on protocol content, many including protocol templates describing essential section headings; however, few resources provide specific guidance on how to actually generate the information required to populate these templates, though some do provide useful advice and instructive examples.

Composed of the protocol development steps, the Concept Protocol Crowd Review Tool, the Protocol Builder, the Study Walk-through Toolkit and a resources section, the Protocol Development Toolkit has therefore been developed to address these gaps. Located within the EDCTP Knowledge Hub, the Protocol Development Toolkit aims to develop a community of practice area for research staff and teams so that they can exchange comments, ask questions and seek help—see Table 2.

**Table 2:** Components of the EDCTP Knowledge Hub: protocol development toolkit

**Protocol development steps—**a step-by-step guide on developing a high-quality health research protocol which provides practical advice and examples on how to develop a protocol, including elements such as writing skills and working collaboratively on the development of a protocol.	
**Concept Protocol Crowd Review Tool**—this short process helps research teams take the first steps in designing a new study by helping them consider what is being asked and what impact the data would have. Here, comments can be sought from the wider research community on the research question being asked, what existing data could be accessed, what outcomes should be measured and how to consider any safety or ethical issues that their study might encounter. This wider community includes the many international partners, collaborators and registered members who contribute to TGHN, representing a full range of research disciplines and roles in global health research [13]. This step is really helpful to initiate the process and guide writing a funding application or guide an institutional review process.	
**Study Walk-through Toolkit**—describes a method to translate a study from a protocol into a successful study. Ideally, this should involve the whole study team to jointly work through every step to identify where training or operating procedures are needed to ensure every time this specific process is safe, accurate and ethical.	
**Protocol builder**—users from LMICs have an opportunity to obtain free access to the SPIRIT Electronic Protocol Tool & Resource (SEPTRE), an innovative, web-based software solution that makes it easier to create, manage and register high-quality protocols for clinical research.	
**Resources**—this section addresses the difficulty a researcher may experience in locating resources using Internet-based searches alone. All of the relevant, open-access, resources retrieved by the specialist software during scoping have been collated to create an extensive collection of nearly 300 resources linked to protocol development which can be searched and filtered depending on their type.	

## The Data Management Portal

Several useful data management toolkits and guides, typically associated with universities or academic libraries, were identified during scoping which all cover similar aspects of data management. *Data management planning*, *data collection*, *data processing*, *data monitoring, data storage and archiving*, *regulation* and *the sharing and re-use of data* tended to be covered in these toolkits in varying levels of detail. However, none appeared to be specifically tailored for low-income settings, and many focused on local policies for data management and sharing. Generally, resources provided an overview of the data management requirements but lacked ‘how to’ guides with advice and examples, or were very detailed and complex, which may be overwhelming to a new data manager.

Composed of the data management steps, a data management plan resource and a resources section, the Data Management Portal has been developed to provide support to researchers to ensure that high-quality data management is fully considered, and planned for, from the outset and implemented throughout the life of a research project (see Table 3).

**Table 3:** Components of the EDCTP Knowledge Hub Data Management Portal

**Data Management Steps**—includes ‘how to’ guides on getting started with data management, from writing a data management plan to designing case report forms and choosing a data management system. ‘Prepare to share’ steps are included throughout the resource, combined with sections on how to organise, process, store, protect and monitor data.	
**Data management plan**—the data management plan templates from 12 major institutions and funders were collated to produce a list of questions which should be considered when creating a data management plan.	
**Resources**—extensive collection of over 600 resources linked to data management which can be searched and filtered depending on their type.	

## Data Sharing Toolkit

Overall, the participants at the Data Sharing Workshop asked for a comprehensive training that ‘starts from scratch’, as they felt that many people would lack the basic skills and knowledge to navigate the process from the beginning. They argued that they would not be able to make sound decisions about how to prepare and share data, unless they have the technical skills necessary to process the data, alongside clear guidelines describing what is required of them. Several examples were given during the workshop; for instance, one of the participants said they could not choose between *open access* and *controlled repository* as this was the first time they had heard that there was such a choice: they would need the options explained in detail first. Others felt that their technical skills would not allow them to prepare the data to a high enough standard. Generally, there was also apprehension around the legalities of data sharing, understanding funder requirements and adequately fulfilling them.

Developed to address these perceived gaps, the Data Sharing Toolkit covers various aspects of the process of sharing research data. This includes how to choose a suitable repository using the Repository Finder; how to organise research data and prepare it for sharing including documentation file; how to navigate copyright, consent and permissions; and how to actually deposit data, with practical examples such as README files and the repository submission process, all in the data management basics and data sharing steps sections (see Table 4).

**Table 4:** Components of the EDCTP Knowledge Hub Data Sharing Toolkit

**Data management basics**—explanations covering different aspects of working with data: from a list of free version control tools to explanation of metadata and real-life examples of data problems encountered by data managers handling clinical data.	
**Data sharing steps**—a step-by-step guide on how to organise and document data, the copyright, consent and permissions issues a user might need to navigate and how to deposit data with practical examples. This section also includes a section on data ownership and reuse, to address the limited skills and resources in this area.	
**Repository finder**—an online decision tree tool to help users select an appropriate repository for their health research data.	
**Resources**—an extensive collection of nearly 200 resources linked to data sharing which can be searched and filtered depending on their type.	

We also have seen that there is little evidence to show that research teams from LMICs are using data sharing repositories to access data themselves. We see this as an important issue that further fuels the real and perceived inequity in health data sharing, which needs to be addressed.

## Conclusions

We have set out to make the necessary skills and knowledge for writing protocols, good data management and data sharing discoverable and easy to use by research teams in low-resource settings, where such support, training and guidance are largely inaccessible.

Whilst the EDCTP Knowledge Hub has primarily been developed to support current and potential EDCTP grant holders in running scientifically sound and ethical studies, it is designed to be open and accessible for all researchers. We aim for these tools and guidance to ensure that research teams can design quality protocols that ask the right question and then measure the right outcome that is acceptable to their community and appropriate for the local clinical setting. These resources should enable data management plans to be written that will ensure reliable and appropriate analysis and present a set of credible and high-quality data, ready for sharing, that comply with existing data standards and good practices.

The EDCTP Knowledge Hub is intended to build on existing initiatives aimed at promoting African scientific leadership and excellence and to complement the significant progress made in recent years to support the development of individual and institutional capacity and research outputs that change the management, prevention and treatment of poverty-related diseases. For example, the volume and citation impact of scientific papers from sub-Saharan Africa has increased since 2003, and papers on poverty-related diseases have become increasingly available as open access publications [25, 26]. Additionally, the collaborative research between Europe and sub-Saharan Africa increased, illustrating the benefit of collaborative research on poverty-related diseases [25]. However, this international collaboration advantage seems to be region-specific, with increased impact for European-wide and European-sub-Saharan African collaborations, whilst a decrease in research impact has been observed for collaborations confined to sub-Saharan Africa, with further verification of these findings and the underlying factors required [26]. Future capacity development activities in sub-Saharan Africa should take these findings into consideration and the EDCTP Knowledge Hub is one such effort that can help to level the playing field and foster long-term collaborative research based on equity between collaborators in the Global North and Global South [10].

There are therefore still important gaps that this and other work have highlighted. In particular, we have learnt that writing standard operating procedures and training plans is a challenge for many teams. Both these elements would help secure reliable and successful studies. By providing access to resources and tools to facilitate the development of high-quality protocols, such as the SEPTRE tool and resource, we hope to also address challenges around publishing well-written, comprehensive study protocols, thus promoting dissemination to reduce publication bias and improve reproducibility.

Perhaps the most important inequity we must highlight through this work is that researchers in LMICs are not accessing data from repositories. If more shared data from wealthy regions was accessed by those in low-resource settings, this would serve to address the current perception that researchers in LMICs have, namely, that sharing their data will not benefit them, and they are just being asked to ‘give it away’. In order to maximise the many potential benefits of data sharing and reuse, we need to create a situation where research teams in the Global South have the skills and knowledge to tangibly and fairly benefit from data sharing as much as those in the wealthier Global North. Therefore, another key gap that remains is supporting researchers in LMICs with accessing shared data to answer new questions that can bring new evidence to support their research, promote collaborations, aid transparency and increase confidence and trust in clinical research data. Ultimately, we need to have and promote open science by supporting equitable data sharing and balancing the benefits of this, being mindful of the multifaceted nature of data sharing, the legitimate interests that need to be protected and the public trust that needs to be built to achieve this.

## Supplementary Information

**Additional file 1.** Process for identification of existing tools and resources and gap analysis.

**Additional file 2.** Search terms used for existing resource identification: Data sharing and data management.

## Data Availability

All data generated or analysed during this study are included in this published article [and its supplementary information files].

## Abbreviations

EDCTP: European & Developing Countries Clinical Trials Partnership
TGHN: The Global Health Network
LMICs: Low- and middle-income countries
