# Supplementary material for: The European & Developing Countries Clinical Trials Partnership (EDCTP) Knowledge Hub: developing an open platform for facilitating high-quality clinical research
Source: Trials. 2022 May 7;23:374. doi: 10.1186/s13063-022-06311-y (PMC9077850; doi:10.1186/s13063-022-06311-y)
Supplement: Supplementary file 1 — Additional file 1. Process for identification of existing tools and resources and gap analysis. [file 13063_2022_6311_MOESM1_ESM.docx]

**Additional File 1:** Process for identification of existing tools and resources and gap analysis

The example below shows the steps taken in order to assess the tools and resources that already exist around protocol development and establish the true gaps. A similar process was undertaken for both data sharing and data management, the search terms utilised for these areas are described in [Additional File 2].

**Protocol development: Process for identification of existing tools and resources and gap analysis**

A three-stage strategy was employed:

- *Stage 1:* Identification of existing resources, with a three-step approach (outlined below).
- *Stage 2:* Quality assessment of all resources identified in Stage 1.
- *Stage 3:* Survey/discussions with members of The Global Health Network/key informants.

**Stage 1: Identification of resources**

This was a three-step approach and included:

1. A general internet-based search.
2. An initial scoping of grey literature which included use of the snowballing method to identify additional sources (such as regulatory guidance documents, and published books such as the Field Trials in Developing Countries Toolkit).
3. Targeted search, drawing on certain organisations e.g. CONSISE, ISARIC etc.

***General internet-based search [Step 1]***

The purpose of this comprehensive audit was to identify all publicly available training courses, learning materials (e.g. templates, standard operating procedures (SOPs)/guidance documents), regulations, guidelines and educational videos, primarily on protocol development in low-resource settings for health researchers. A specialist software (DEVONagent Pro) was used to carry out an internet-based meta-search querying multiple search engines (via “plugins”) simultaneously. We included: Bing, Google, Google (PDF), Yahoo!, Google Video, YouTube, Medline Plus, PubMed, WebMD, Google Scholar (Articles), and JSTOR. Up to 200 results per plugin were collected during each search. To begin the process, terms in Table 1 were employed, followed by a series of more in-depth four-level queries (Table 2). The search terms used for data management and data sharing are shown in Appendix B.

Table 1. List of search terms used during the initial scoping exercise:

| Search terms |
| --- |
| Creating a protocol |
| Guidance for protocols |
| How to write a protocol |
| Protocol development in clinical research |
| Protocol development process |
| Protocol development tool |
| Standard research protocol |
| Writing a protocol |

Table 2. List of search terms used during the scoping exercise. Combinations of these keywords yielded 234 search queries. Phrasing examples: (i) using the first set of terms: protocol AND (disease* NEAR/10 poverty) AND (clinical NEAR/5 research OR trial) AND educational; (ii) using the last set of terms: protocol AND (low NEAR/2 resource*) AND vaccine AND seminar*.

| First part of the search term | Second part of the search term | Third part of the search term | Fourth part of the search term |
| --- | --- | --- | --- |
| protocol | (disease* NEAR/10 poverty) | (clinical NEAR/5 research OR trial) | educational |
|  | health | device | "formal guidance" |
|  | (low NEAR/2 resource*) | diagnostic | guid* |
|  |  | drug | learning |
|  |  | Phase | "Standard Operating Procedures" |
|  |  | vaccine | SOP* |
|  |  |  | tutorial* |
|  |  |  | template* |
|  |  |  | tool* |
|  |  |  | (user NEAR/5 guide*) |
|  |  |  | video* |
|  |  |  | lecture* |
|  |  |  | seminar* |

Overall, 243 search queries were run (9 based on Table 1 and 234 based on Table 2). While fourteen searches did not return any results, in total, the searches returned 5,120 unique results. These were then individually assessed by a clinical specialist in order to ensure their relevance to the project and quality, any items identified as unsuitable were removed (Stage 2).

***Search of grey literature [Step 2]***

This scoping exercise concluded that ICH GCP is the main text indicated, which is perhaps not surprising as researchers are directed to adhere to these guidelines for publication. To date, useful supporting documents have been identified from “The Medical Research Council (MRC)” and 3rd Edition of "Field Trials of Health Interventions: a Toolbox" http://teg.lshtm.ac.uk/.

***Targeted, organisational search [Step 3]***

We undertook a targeted internet-based search reviewing examples of shared protocols from expert groups such as CONSISE (Consortium for the Standardization of Influenza Seroepidemiology) and ISARIC (International Severe Acute Respiratory and Emerging Infection Consortium).

*Referenced resources* – during the quality assessment of the resources identified in Stage 1 any further note-worthy resources mentioned within the material were retained.

**Stage 2: Quality assessment & gap analysis**

A two-step approach was employed:

1. Resources identified in Stage 1 were quality assured by reviewing their content and assessing their suitability for, and usefulness to, the target audience. The quality assessment was carried out by a clinical specialist.
2. Efforts were made to further explore perceived ‘gaps’ in the content identified in Stage 1 and will be addressed in the Protocol Development Area on the EDCTP Knowledge Hub Platform.

**Quality assurance [Step 1]**

The suitability of materials was determined initially from the summary information, and then subsequently by exploring the main content. This task was undertaken by members of The Global Health Network, and an independent clinical specialist, who reviewed the final list of materials. For the general internet-based search (Stage 1, Step 1), resources were first reviewed for relevance to the audit remit. Those resources clearly unrelated to clinical research could be excluded based on summary information e.g. music videos, materials relating to internet-based protocols or clinical care protocols. For the remaining resources we used specific inclusion and exclusion criteria to determine suitable resources.

**Inclusion criteria**

The specific inclusion criteria for materials were as follows:

- Materials must fit into at least one of the pre-determined themes listed in Tables 1 and 2.
- Materials that are open access.
- Materials that are free.
- Materials providing detail of the protocol development process for a clinical study.
- Materials that are from a reputable organisation or course provider e.g. FutureLearn,
- Materials can be in a range of formats e.g. PowerPoint presentations.

**Exclusion criteria**

The specific exclusion criteria for materials were as follows:

- Materials that do not fit into at least one of the pre-determined themes listed in Tables 1 and 2.
- Materials that are not open access.
- Materials that have a cost implication/charge an access fee.
- Materials that are not from a reputable organisation or course provider e.g. an unaccredited blog or materials based on a personal or biased opinion.
- Materials that are not in a suitable format.
- Materials that are too specific to an organisation, such as internal training that includes organisation specific approval processes.
- Materials that are identified in more than one search will be listed once.

**Exploration of perceived ‘gaps’ in content [Step 2]**

In order to identify the gaps in protocol building guidance the main content of each resource retained following Step 1 was reviewed in order to assign a resource type and keywords to describe the resource (Table 3).

Table 3: List of keywords and resource type categorisation following review:

| Keywords (one or more assigned) | Type (one assigned) |
| --- | --- |
| Data management | Bulletin/Article |
| Development | e-Learning Course |
| Ethics & consent | Guide |
| Good clinical practice | Learning materials |
| Implementation | Lecture/Seminar (recorded structured presentation) |
| Planning | Report/Brief - technical writing |
| Quality assurance | Short educational video (anecdotal, overview, short etc.) |
| Regulation | Slides/Presentation (if not in video format) |
| Related documents | Toolkit |
| Review process |  |
| Safety reporting - adverse events etc. |  |
| Standard Operating Procedure (SOP) |  |
| Study procedures |  |
| Template |  |

Each resource was allocated one or more ‘keywords’ and one ‘type’ categorisation. These data were used to establish in which areas there were gaps and what type of resources exist allowing the resources made available on the EDCTP Knowledge Hub to be tailored to fill these gaps.
