# Supplementary material for: The European & Developing Countries Clinical Trials Partnership (EDCTP) Knowledge Hub: developing an open platform for facilitating high-quality clinical research
Source: Trials. 2022 May 7;23:374. doi: 10.1186/s13063-022-06311-y (PMC9077850; doi:10.1186/s13063-022-06311-y)
Supplement: Supplementary file 2 — Additional file 2. Search terms used for existing resource identification: Data sharing and data management. [file 13063_2022_6311_MOESM2_ESM.docx]

**Additional File 2:** Search terms used for existing resource identification: Data sharing and data management

**Data sharing**

To begin the process, terms in Table 1 were employed, followed by a targeted organisation search (Table 2) and a series of more in-depth queries (Table 3).

Table 1: A full list of the specific search terms used for both Google and Google videos during the first-round of resource identification around data sharing. Specific examples of phrasing would be “data access in health research online training” etc.

| First part of the search term | Second part of the search term | Third part of the search term |
| --- | --- | --- |
| Data access | *‘in Health Research’* | *‘online training / course’’*  *[e.g. videos (Lecturio)]* |
| Data archiving |  |  |
| Data catalogue |  |  |
| Data citation |  |  |
| Data curation |  |  |
| Data discovery |  |  |
| Data integrity (+ fixity) |  |  |
| Data mining |  | *‘guidance’*  *[e.g. video (Seminars)]* |
| Data mining + data visualisation |  |  |
| Data preservation (+ protection) |  |  |
| Data repository |  |  |
| Data reproducibility |  |  |
| Data retention |  |  |
| Data reusability |  |  |
| Data security |  | *‘resources’*  *[e.g. templates, guidelines, training manuals, handbooks, handouts, SOPs.]* |
| Data stewardship |  |  |
| Data sharing |  |  |
| Data storage |  |  |
| Data transparency |  |  |
| Data usage |  |  |
| Data usage tracking |  |  |
| Deposit(ing) data |  |  |
| Digital data storage |  |  |
| Digital preservation |  |  |
| Digital repository |  |  |
| FAIR data |  |  |
| FAIR guidleines |  |  |
| FAIR principles (findable, accessible, interoperable and re-usable) |  |  |
| FAIRSharing |  |  |
| FORCE11 (Future of Research Communication and E-scholarship) |  |  |
| Machine-readable data |  |  |
| Metadata |  |  |
| Metadata harvesting |  |  |
| Metadata publishing (publication) |  |  |
| Open access data |  |  |
| Open data |  |  |
| Open research data |  |  |
| Open source data |  |  |
| Storing data |  |  |

***Organisational search***

A targeted internet-based search was undertaken to identify organisations and institutions that hosted further online information related to data sharing and data repositories. This search included reputable organisations with a vested interest or established reputation relevant to the field of data sharing within health research (e.g. funders, journals, academic institutions, data hubs etc.), as well as those identified from the landscaping exercise and other available literature (Table 2).

Table 2: This table illustrates the following organisations which were scoped, as a result from any internet-based searches and also by recommendation.

| - Digital Curation Centre (DCC) |
| --- |
| - Distributed Data Curation Center at the Purdue University Libraries |
| - Elsevier: https://researcheracademy.elsevier.com/# |
| - EU |
| - Food and Drug Administration (FDA) |
| - Figshare: https://figshare.com/articles/Whitepaper_Practical_challenges_for_researchers_in_data_sharing/5975011 |
| - Foundation Merieux |
| - Gates |
| - Harvard |
| - Institute of Pharmaceutical Science |
| - Institute Pastuer |
| - Institute of Tropical Medicine (ITM), Antwerp |
| - Journals |
| - London School of Health and Tropical Medicine (SHTM) |
| - Medical Research Council (MRC) |
| - NHS |
| - National Insitute of Health (NIH) |
| - NYU Health Sciences Library: http://hslguides.med.nyu.edu/data_management |
| - PARTHENOS “Pooling Activities, Resources and Tools for Heritage E-research Networking: <http://training.parthenos-project.eu/sample-page/manage-improve-and-open-up-your-research-and-data/> |
| - PharmaSchool |
| - Public Health Schools |
| - Relief web: <https://reliefweb.int/training?cost=free#content> |
| - Sheffield (has a useful table (all three tabs)) <https://www.sheffield.ac.uk/library/rdm/repositories#tab00> |
| - Swiss Tropical Medicine |
| - Universities e.g. <https://mantra.edina.ac.uk/> |
| - Wellcome |
| - WHO |
| - UK Data Archive |
| - University of Oxford website <http://researchdata.ox.ac.uk/> |

Table 3: This table illustrates the search terms included in the second-round of resource identification around data sharing using DEVONagent Pro as guided by the ‘gaps’ presented in the results from Stage 1*.*

| First part of the search term | Second part of the search term | Third part of the search term |
| --- | --- | --- |
| data security | “in health research” | guidance |
| FAIRsharing |  |  |
| digital repository |  |  |
| data usage |  |  |
| FORCE11 (Future of Research Communication E-scholarship) |  |  |
| open research data |  | resources |
| Metadata publishing (publication) |  |  |
| data storage |  |  |
| metadata |  |  |
| digital data storage |  | online training/course |
| data usage tracking |  |  |
| open data |  |  |
| data sharing |  |  |

**Data Management**

As with the protocol development and data sharing examples, to begin the process, terms in Table 4 were employed, followed by a series of more in-depth queries (Table 5).

Table 4. A full list of the specific search terms used for both Google and Google videos during the first-round of resource identification around data management. Specific examples of phrasing would be “Health research risk assessment” etc.

| First part of the search term | Second part of the search term |
| --- | --- |
| Health research | risk assessment |
| Clinical research | software |
|  | grant |
|  | study admin system |
|  | administrative database |
|  | randomisation |
|  | data management |
|  | CRF |
|  | audit trail |
|  | case report form* |
|  | query management |
|  | central monitoring |
|  | site visit* |
|  | safety |
|  | reporting |
|  | database lock* |
|  | archiv* |
|  | back up |
|  | disseminati* |
|  | data verification |

Table 5: This table illustrates the search terms included in the second-round of resource identification around data management using DEVONagent Pro.

| First part of the search term | Second part of the search term | Third part of the search term |
| --- | --- | --- |
| health research | admin database |  |
| clinical research | admin database |  |
| "case report form" | design |  |
| clinical data management | course |  |
| clinical data management | application |  |
| crf | design |  |
| clinical data management | training |  |
| clinical data management | guidance |  |
| clinical data management | resource* |  |
| health research | data accuracy |  |
| health research | data aggregation |  |
| health research | data analysis |  |
| health research | anonymi*ation |  |
| health research | data attributes |  |
| health research | data audit |  |
| health research | data basics |  |
| health research | data checks |  |
| health research | data cleaning |  |
| health research | data coding |  |
| health research | data collection |  |
| health research | form design |  |
| health research | data dictionary |  |
| health research | data documentation |  |
| health research | data entry |  |
| health research | data fundamentals |  |
| health research | data governance |  |
| health research | data identifiers |  |
| health research | data integration |  |
| health research | data integrity |  |
| health research | data logic |  |
| data management | best practices | health research |
| data management | budget | health research |
| data management | case studies | health research |
| data management | committee | health research |
| data management | equipment | health research |
| data management | good clinical practice | health research |
| data management | lifecycle | health research |
| data management | lmic | health research |
| data management | oversight | health research |
| data management plan | health research |  |
| data management practices | health research |  |
| protocol violat* | health research |  |
| data management | regulation | health research |
| data management | skills | health research |
| data management | software | health research |
| data management | sop | health research |
| data management | staff | health research |
| data management | standard operating procedures | health research |
| data management | system design | health research |
| data management | system validat* | health research |
| data management tools | health research |  |
| data monitor* | health research |  |
| data organisation | health research |  |
| clinical data management (pdf) | |  |
| clinical data management (docx) | |  |
| clinical data management (doc) | |  |
| data organisation | health research |  |
| data ownership | health research |  |
| data processing | health research |  |
| data quality | health research |  |
| data range checks | health research |  |
| data recording | health research |  |
| data reports | health research |  |
| data source | health research |  |
| data standard* | health research |  |
| data structure | health research |  |
| data system* | health research |  |
| data validat* | health research |  |
| data visualisation | health research |  |
| database | health research |  |
| version control | health research |  |
| dataset* | health research |  |
| digital data | health research |  |
| double data entry | health research |  |
| (electronic case report form OR electronic crf ) | health research |  |
| electronic data management tool* | health research |  |
| exploratory data analysis | health research |  |
| fair principles | health research |  |
| fieldname* | health research |  |
| medical coding dictionar* |  |  |
| interim analysis | health research |  |
| open access | health research |  |
| patient questionnaire design | |  |
| research data management | health research |  |
| rdm | health research |  |
| source document verification | health research |  |
| structured data | health research |  |
| unstructured data | health research |  |
